# The Danger of Flowering and Fruit-Bearing Plants in Sleeping Apartments

**Published:** 1876-07

**Authors:** 


### The Danger of Flowering and Fruit-Bearing Plants in Sleeping Apartments.

The volatile matters which are exhaled from fruits and flowers possess, in a high degree, the power and tendency of absorbing the oxygen in the surrounding air, thus creating a corresponding quantity of carbonic acid.

By this reaction the salubrity of such air for those who breathe it is injured in two ways, both by the abstraction of oxygen and by the addition of carbonic acid. Since this fact is but too little known, and still less regarded, it ought from time to time to be mentioned, and instances given of fatal results from the neglect of its manifest teachings.

A gentleman suffered from severe headache, and felt such weakness that he could scarcely keep awake. He notlced some hyacinths in the chimney, which he removed and opened the window, when his simptoms all became less severe; but it was some days before his head became entirely clear. Another man had the unlucky idea to construct a bower of oleander branches in the chamber in which he slept. The next morning he was found dead in bed. A grocer and his servant slept together in a room in which were three chests filled with oranges, and they, also, were found dead in the morning. A clerk in a warehouse, who performed the duties of night-watchman, used a sack containing saffron as a pillow, with a like fatal result. — DR. REITTER, in *Wiener Med. Press*, No. 43, 1876.

